# Expanding Access to Malaria Diagnosis through Retail Shops in Western Kenya: What Do Shop Workers Think?

**DOI:** 10.1155/2013/398143

**Published:** 2013-05-21

**Authors:** Andria Rusk, Catherine Goodman, Violet Naanyu, Beatrice Koech, Andrew Obala, Wendy Prudhomme O'Meara

**Affiliations:** ^1^Duke Global Health Institute, Trent Hall, Durham, NC, USA; ^2^London School of Hygiene and Tropical Medicine, London, UK; ^3^Moi University School of Medicine, College of Health Sciences, Nandi Rd, Eldoret, Kenya; ^4^Webuye Demographic Surveillance Site Scientific Steering Committee, Eldoret, Kenya; ^5^Duke University School of Medicine, Division of Infectious Diseases, Durham, NC, USA; ^6^Moi University School of Public Health, College of Health Sciences, Nandi Rd, Eldoret, Kenya

## Abstract

*Background*. The common symptoms of malaria reduce the specificity of clinical diagnosis. Presumptive treatment is conventional but can lead to overdiagnosis of malaria, delay of appropriate treatment, overprescription of antimalarials, and drug resistance. Routine use of diagnostic tests can address many of these concerns. Though treatment is often procured from retailers, there is low availability of rapid diagnostic tests for malaria (MRDTs), a simple, inexpensive, and accurate diagnostic solution. We know little about the challenges to expanding access to diagnostics through these outlets. *Methods*. To understand the perceptions of the benefits and challenges to selling rapid diagnostic tests for malaria, we conducted focus group discussions with antimalarial retailers who serve the residents of the Webuye Health and Demographic Surveillance Site in western Kenya. *Results*. Medicine retailers perceived MRDTs to be beneficial to their customers and businesses but also included cost, fear of the tests, risks of self-treatment, and regulatory concerns among the challenges to using and selling MRDTs. *Conclusion*. MRDTs represent a viable approach to increase access to malaria diagnostic testing. Medicine retailers are eager for MRDTs to be made available to them. However, certain challenges remain to implementation in retail outlets and should be addressed in advance.

## 1. Background

Malaria contributes significantly to the global burden of disease, causing an estimated 0.5 to 2.5 million deaths each year [1]. To reduce the disease burden, international organizations advocate treatment for malaria to be administered within 24 hours of the first symptoms of the disease [2]. However, several signs and symptoms associated with malaria are shared by a host of other diseases, such as influenza and pneumonia [3]. As a result, clinical diagnosis has poor specificity for malaria [4], often resulting in overdiagnosis of malaria, overprescription of costly antimalarials [5], and underdiagnosis or delayed treatment of the true cause of fever [3, 6].

Improving access to accurate parasitological diagnosis is now of critical concern for malarial control [7]. Microscopic examination of stained blood films remains the gold standard for diagnosing malaria, but microscopy requires trained laboratory staff, costly equipment, a constant supply of reagents, and quality control and supervisory systems [8, 9]. Compared to microscopic testing, rapid diagnostic tests for malaria (MRDTs) are easy to use, do not require any specialized equipment, and are relatively inexpensive, with a retail price of between $0.58 and $1.15 [10, 11].

Several studies have shown that implementation of MRDTs in health facilities can reduce overprescription of antimalarials [8, 12], while supporting appropriate treatment for those with positive test results. However, health facilities are not the only source of treatment for febrile illness, or even the main source in many settings. Across sub-Saharan Africa, private for-profit medicine outlets account for between 33 and 95% of the market share of antimalarial sales volume [11]. In Kenya, the private for-profit market share is between 50 and 80%, and medicines purchased at retail drug outlets were the first or only treatment for 34–42% of childhood fevers [13]. However, a recent study showed that in an area of relatively high malaria endemicity, only 56% of clients seeking antimalarials in the retail sector tested positive for malaria [14]. Improving targeting of ACTs to patients with malaria is likely to require expanding access to diagnostics through drug outlets such as retail shops, chemists, and pharmacies, where treatment for febrile illness is often first sought [15, 16]. 

Retail drug shops have limited capacity to offer microscopic testing (MST) for malaria [2, 11, 17]. This is in part because the Kenya Pharmacy and Poisons Board places certain requirements on shops that offer diagnostic services including requiring that specifically trained staff conduct all diagnostic tests. The board also requires that all retail drug outlets be registered with the board, that the operator of the outlet be licensed by the board, and that certain medicines be dispensed under the supervision of a certified pharmacist or pharmaceutical technologist [18]. In reality, very few retailers meet these requirements and therefore very few are formally registered. 

The artemisinin combination therapy (ACT), artemether-lumefantrine (AL), is the first-line antimalarial in Kenya and is designated as a prescription-only medicine, although it is readily available over the counter with a prescription. Since 2010, the Kenyan National Malaria Treatment Guidelines dictate that all suspected malaria cases should receive parasitological confirmation before treatment with AL, but in practice, availability of diagnosis is restricted to a fraction of public health facilities and is practically nonexistent in the retail sector.

Kenya participated in the Global Fund's ACT subsidy programme, the Affordable Medicines Facility-malaria (AMFm), since 2010, leading to substantial falls in the price of AL in the private sector [19, 20] and unofficially legitimizing over-the-counter sales of AL. The Global Fund has now indicated that the transition to the next phase of AMFm will include the option to use money from Global Fund grants to subsidize MRDTs in the retail sector [21]. Kenya will now have to decide whether to permit sale of MRDTs in the retail sector given the success of AMFm in order to reach the large number of patients who currently purchase AL over the counter without a diagnostic test. 

 In summary, in Kenya, as in much of sub-Saharan Africa, medicine retailers have an important role in the clinical diagnosis and treatment for fevers. MRDTs are a simple and affordable alternative that could be used effectively in retail drug outlets without requiring capital investment and specialized staff, and implementing MRDTs in the private retail sector could considerably improve the targeting of ACTs. Despite the reported success of MRDT implementation in the public health sector, very little is known about implementation in the private sector [17, 22–24]. Understanding the perceptions of medicine retailers would be critical to successful implementation of MRDTs in this environment, particularly as Kenya is deciding whether to legalize and/or subsidize MRDTs in the retail sector as part of its malaria control strategy. We therefore conducted a study using focus group discussions with retailers in a rural district in Kenya to gain an understanding of their perspectives of the benefits and challenges to using and selling rapid diagnostic tests for malaria. 

## 2. Methods

### 2.1. Study Area and Sample

The study was conducted in the Webuye Health and Demographic Surveillance Site (WHDSS) in the Bungoma East District of Kenya's Western Province. The study area's location, demographics, economy, and healthcare system have been described in detail elsewhere [25].

The study population included all private for-profit retail locations that sold antimalarials and were within or accessible to those living in the WHDSS. General shops that sold household goods were excluded. Retail outlet locations had been identified and mapped by recorded GPS coordinates during prior research [20]. Any new retail locations that had opened since the last mapping activity were also invited to participate. Ninety-one outlets were identified during this canvassing activity.

**Figure 1:**
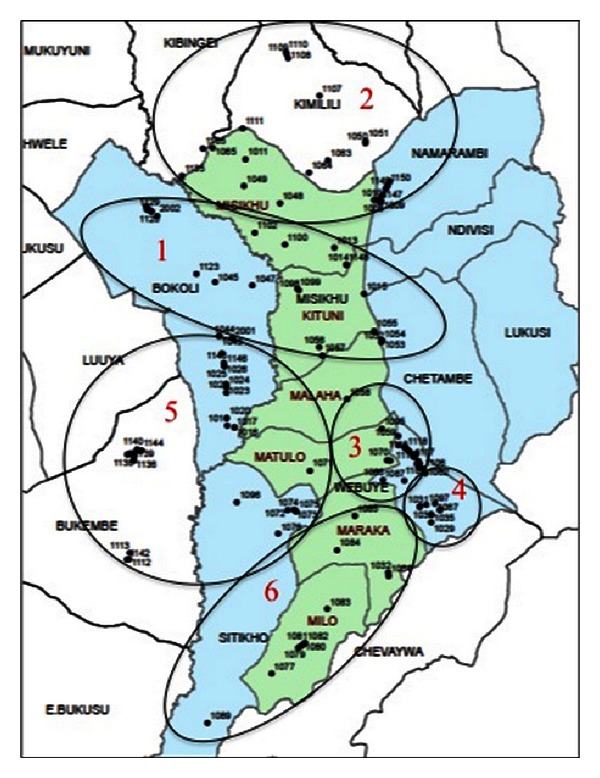
Map of study area including numbered boundaries of each focus group catchment area.

Using the maps as a guide, discussions were held in locations as convenient as possible to the retailers in those shops, taking into consideration road conditions, river barriers, and natural gathering places such as markets and community centers (see Figure 1 for the clustering strategy). The resulting locations included 4 discussions at rural locations (held in Lugulu, Misikhu, Bukembe, and Milo) and two peri-urban discussions, both held in Webuye Town at the DSS field office. 

### 2.2. Data Collection

The focus group discussions (FGDs) were conducted in July 2011 by a female social scientist trained in FGD moderation and fluent in both Kiswahili and English. The discussions began with a brief questionnaire focused on participant education and health qualification level, previous experience with malaria diagnosis and treatment, and knowledge of specific diagnostic methods. Following this, the discussion explored experiences with malaria diagnosis and treatment, retailer relationships with customers, a demonstration of a rapid diagnostic test for malaria, and perceptions of the test, its feasibility, profitability, and potential role in the diagnostic and treatment process. Specific questions were asked regarding treatment patterns in retail drug outlets, and how the introduction of MRDTs might influence these patterns. Of the 91 outlets identified, 61 participants attended the focus groups, with a median group size of 10.

 The FGD guide and questionnaire were pretested in Eldoret, Kenya, revised, and translated into Kiswahili. Revisions made following the pilot increased clarity, relevance and reduced repetition. The moderator was trained on the implementation of this specific discussion guide as well as on the study protocol and objectives.

 The MRDT selected for the demonstration was the ICT malaria Pf cassette by ICT Diagnostics, which meets the recommended selection criteria for malaria rapid diagnostic tests as published by the World Health Organization [26]. A laboratory technician employed by the Webuye District Hospital performed the demonstration. 

### 2.3. Data Entry and Analysis

Focus group discussions were recorded with digital audio recorders and translated into English by the research team. Hand-written notes captured during the discussions were also translated into English. Both sources were entered and coded using QSR NVivo 9 software. Initial codes were developed based on the existing literature. After these data were examined, subsequent codes were developed from themes that emerged during data review. Data were coded into these themes and then analyzed for similarity, frequency, strength, and relationship. These themes were vetted with the research team, project collaborators, and local members of the Webuye DSS team to gain more insight into the significance of the emerged themes. A second analysis was then conducted, incorporating these additional findings, and drawing new comparisons between the more relevant themes.

### 2.4. Ethical Considerations

Ethical approval was granted from Moi University Institutional Research and Ethics Committee in Eldoret, Kenya, and the Duke University Institutional Review Board in Durham, NC, USA. All study participants were informed of the objectives of the study in the written invitation as well as prior to participation in the discussion. Verbal consent was obtained from all participants in the study. Verbal consent was also obtained from all participants to voice recordings of the discussion, manual notation of discussion subject matter, nonverbal behavior, and environmental content. Study participants received light refreshments at the close of the discussion, and reimbursement for their travel expenses (~3.5 USD per participant).

## 3. Results

### 3.1. Characteristics of Medicine Retailers

More than half the study population was female (59%) and between the ages of 21 and 40 (66%). Most participants had received training in the field of pharmacy (44%) or nursing/midwifery (18%) with two clinical officers (3%) and one participant without any formal health training. Most participants reported completing secondary school (41%), with others reporting some education above secondary school (39%). However, the level of education reported did not always align with reported health training. One clinical officer stated to have received only a primary school education, and 5 out of 11 pharmacists and 8 out of 17 nurse/midwives reported no education above secondary school. Participant characteristics by focus group are covered in Table 1.

### 3.2. Perceived Benefits of Selling MRDTs in Retail Drug Outlets

Though most medicine retailers were not familiar with rapid diagnostic tests for malaria (84%), after the demonstration, many of them felt it would be a beneficial product.

*“If it can be easily available, it can prevent this issue of giving patients drugs without knowing the exact disease because somebody can come to the shop explaining those symptoms related to malaria, but if you have the kit, you can test the patient and confirm there instantly.” (FGD 2, Webuye)*

Several specific benefits to implementing MRDTs in retail outlets were identified by retailers, including furthering retailer expertise and reducing malaria incidence in the area. Four major benefits emerged in the analysis which are elaborated below: MRDTs may attract more business to drug outlets, MRDTs may save time and money for both customers and retailers, they may increase access to malaria diagnostics and thereby appropriate treatment, and they may increase customer and retailer confidence in diagnosis and treatment.

### 3.3. MRDTs May Bring Business to Shops

Retailers anticipated that selling MRDTs in their outlets would help them retain customers that they would have previously referred to hospitals or laboratories for testing. They felt they could even bring in new customers by offering diagnostic services, and that this could improve an outlet's reputation making them more attractive than their competitors.

*“I think you will also be getting direct patients, not those who come with prescriptions. You actually create your own patients. After testing, now you have your clients. Like me, I will not be referring. Sometimes you send a client for testing and she/he goes away. So it's a benefit.” (FGD 5, Bukembe)*

### 3.4. MRDTs May Save Time and Money

Retailers felt that selling rapid diagnostic tests in drug outlets would prevent customers from needing to travel to public health facilities for testing, saving customers the time and cost of travel. Patients would also be able to save time by avoiding the long queues and additional costs associated with public health facility visits.

Retailers perceived that using rapid tests for malaria would save them money as well, since it is cheaper for them than microscopy, in terms of equipment price and staff training. Only 7% of medicine retailers had ever performed MST in their outlets. Performing microscopic testing can be challenging for retail drug outlets due to the cost of equipment, training personnel, and necessary experience to read slides accurately [27].

Conducting MST is also a laborious and time-consuming process. The simplicity and ease of use of MRDTs was seen as a benefit by participants, specifically over microscopic testing, which may lead to an increase in use of diagnostic testing by retailers. It was discussed that rapid tests also deliver a definitive diagnosis quickly, allowing retailers to serve more customers in less time.

*“I think it is a very nice one because now you do not need to go for a microscope and it's cheaper. The microscope, the machine is very expensive, but for this one, if you buy at Ksh.50 ~USD $0.58, you can afford Kshs.500 ~USD $5.75 per week and test those clients. Also, everybody can test provided the instructions are followed well.” (FGD 5, Bukembe)*

### 3.5. MRDTs May Increase Appropriate Treatment and Access to Malaria Diagnostics

Retailers saw MRDTs as a way to increase access to malaria diagnostics. Since their outlets are open more often than public health facilities and outlets service rural areas with limited access to public facilities, offering MRDTs in retail locations would increase access to diagnostics to these communities. They also saw offering testing in their outlets to be more convenient and less costly for their customers, also increasing access since this may encourage more customers to take advantage of testing services. 

Retailers felt that increasing the use of malaria diagnostics would also increase the appropriateness of treatment. Retailers said this was significant to them, and that they felt it was important to treat patients accurately.

*“When you test, it can help you to give the right drugs to the patient.” (FGD 3, Lugulu)*

*“What matters most is the test, to know exactly what you are treating. That's the most important thing.” (FGD 3, Lugulu)*

### 3.6. MRDTs May Increase Confidence in Diagnosis and Treatment

Retailers felt that having access to definitive diagnosis could increase a customer's confidence in the accuracy of that diagnosis, and in the treatment they receive. They may be more likely to accept the treatment recommended by the retailer, or to purchase alternative medications in cases with a negative test result. Retailers also felt that customers may have more confidence in MRDTs than in laboratory tests.

*“I think the patient will be comfortable because she/he will just look at the results. Maybe in the lab, the person may not be able to see the results.” (FGD 1, Webuye)*

Retailers also appreciated avoiding uncertainty in their diagnoses by using the tests.

*“I think there will be no more guess work. I will be doing something I am sure of and dispensing the right drug.” (FGD 5, Bukembe)*

### 3.7. Perceived Challenges to Selling MRDTs in Retail Drug Outlets

Medicine retailers recognized several challenges to implementing and selling MRDTs in their outlets. Of the concerns mentioned, four challenges were mentioned most frequently and with the most emphasis. They include the costs of MRDTs, customer fears associated with diagnostic testing, the risk of customer self-treatment with the tests, and regulatory problems that providing diagnostic services might cause for retail drug outlets.

#### 3.7.1. The Cost of MRDTs May Dissuade Customers

Retailers perceived that customers may not be able to afford any additional cost beyond that of treatment. There were also concerns that some customers would believe retailers were selling tests only to make more money, and that the cost is being manipulated by retailers to increase profits. It was felt that these issues could be overcome if customers are educated on the use of MRDTs, and the importance of diagnostic testing.

*“Some customers will think we want to squeeze out some money from them. Some will even say, my money was meant for buying drugs not testing. This is especially so among those who do not know. So, a lot of talking and convincing is needed. If good health is given, they will understand.” (FGD 5, Bukembe)*

#### 3.7.2. Customers May Fear MRDTs

Some medicine retailers held the perception that some of their customers may be afraid of getting tested, associating the finger prick necessary for an MRDT with a needle injection. Others felt that their customers may associate the malaria test with an HIV test. Retailers also said that if they started testing with MRDTs, their customers might think the government is secretly testing for HIV.

*“There might be a challenge somewhere, in local areas, we have some people who fear these HIV/AIDS tests, so they might think that now the government is involved in testing malaria but in a real sense they are testing HIV/AIDS.” (FGD 6, Milo)*

#### 3.7.3. MRDTs May Lead to Self-Medication and Reduce Business in Retail Drug Outlets

Several retailers expressed strong opinions against selling rapid tests directly to customers to perform themselves at home. One of these concerns was that customers may test at home and not use the tests correctly, which could lead to incorrect results, but customers may treat themselves at home according to these results. There were fears that this could lead to misdiagnosis or underdiagnosis, and mismanagement of undetected diseases. There were also concerns that testing and treating at home removes the opportunity for the shop worker to sell a nonmalaria drug.

*“RDTs should not be sold to customers because we will have self-medication. Someone could be suffering from something else, and someone may end thinking she/he has been bewitched and maybe it could be another illness so, strictly, RDTs should not be sold to customers.” (FGD 1, Webuye)*

MRDTs do not indicate the severity of malaria infection, and several retailers worried that this may bring harm to children.

*“Like for me, I think it should not be used in children because they are very delicate. Maybe the child has severe malaria and the RDT does not show the severity. So we shall lose our babies.” (FGD 1, Webuye)*

There were also concerns that self-testing may cause outlets to lose business.

*“…some customers are very funny. They may just come and buy and go, because it has some manual inside, so they will start testing at home…They can make our customers disappear.” (FGD 4, Misikhu)*

#### 3.7.4. MRDTs May Create Regulatory Problems for Retail Drug Shops

Medicine retailers expressed concerns that the regulatory bodies overseeing pharmacies and chemists would not allow them to carry out testing in their outlets. There were also concerns that carrying MRDTs would bring the attention of the regulatory board, especially on those locations that are unregistered, and may even require outlets to hire more staff, bringing additional cost to the owners if they wish to conduct testing.

*“In my case, if I carry out such a diagnostic test, I must have a lab technician, and in fact, I am supposed to register the premises. Because if I am found by the P.H.O. public health officer, they can really harass me.” (FGD 4, Misikhu)*

Some retailers, particularly those that are near public health facilities, felt these facilities may prevent the outlets from performing malaria testing.

*“Personally, my shop is located near a dispensary which has got no laboratory. If they get to know that I am now performing RDTs for malaria, they will even threaten to close my shop.” (FGD 6, Milo)*

#### 3.7.5. Acceptance and Adherence

Retailers perceived challenges regarding customer acceptance of and adherence to test results, particularly if results are not in agreement with the customer's desired outcome. They felt that customers who expected a positive result but tested negative for malaria would likely demand antimalarials anyway. This was seen as a particular problem with clients, often mothers with sick children, refusing an alternative diagnosis and even concluding witchcraft was causing the illness.

*“The mother won't accept the results…some will think of supernatural things…like bones, that is, witchcraft. How can the child be sick and there's no malaria?” (FGD 3, Lugulu)*

Participants also anticipated customer resistance in cases with positive test results when the patient does not believe they have malaria.

*“in a chemist, some patients are very rude. They will tell you ‘I do not have malaria' irrespective of the test. ‘How do you give me antimalarials?'” (FGD 1, Webuye)*

Customer resistance to adhering to a test result may put pressure on retailers, making it difficult for them to adhere to results in terms of dispensing appropriate medication as well. However, they did not report seeing the loss of a drug sale as a motive to not adhere to test results. Some said they would dispense antimalarials anyway, and yet others stated that they would put a higher priority on treating their customers well than making a sale, presenting motivations outside of profit maximization as influences on their behavior.

*“To me actually, even if it is business, it is better you heal somebody than getting money for its sake. So to me, I do not see any problems withholding the wrong drugs, so I better treat the patients, not just get money from somebody.” (FGD 3, Lugulu)*

There remained concerns regarding adhering to test results if a retailer feels the test was inaccurate. There were cases cited in which retailers believed a test would not detect malaria, and that in those cases, retailers would not dispense according to test results.

*“Sometimes they test for malaria but it may not show up, but they could be having malaria.” (FGD 1, Webuye)*

## 4. Discussion 

This study explored drug retailer perceptions of the challenges and benefits to using and selling MRDTs in their shops. Retail medicine outlets are an important source of symptomatic diagnosis and treatment for febrile illness. Self-medication with drugs purchased at retail outlets is a common practice [8, 13, 16], and medicine retailers have an important role in the diagnosis and treatment of their customers [23, 25]. There is increasing interest in improving treatment for malaria through the private sector, as exemplified by implementation of the global ACT subsidy through AMFm in 7 countries, including Kenya. The AMFm program expanded access to affordable and effective antimalarials [19] but raised questions about overtreatment of fevers with over-the-counter ACTs [28, 29]. With the high volume of clients seeking antimalarials in the retail sector, it is important to increase access to inexpensive and effective diagnostic testing services to reduce the delay in treating the true cases of febrile illness, and to limit the overprescription of antimalarials. 

While in Africa the expansion of malaria diagnosis in the retail sector has been limited to date to small-scale pilot projects, efforts to increase access to and quality of malaria diagnosis and treatment in the retail sector at a national scale have been underway in Cambodia since 2000. However, despite extensive marketing and training efforts, availability of MRDTs in retail shops remained suboptimal; 49% of shops stocked MRDTS in 2007 but only 21% of patients with recent malaria symptoms were tested [30]. Lessons learned from the Cambodian experience included the importance of conducting qualitative assessments to understand how implementation should be designed and improved. The researchers also underscore the importance of identifying both financial and nonfinancial barriers to using and selling MRDTs, and to address these barriers before implementation. 

There are very few reports of qualitative studies exploring the readiness of retail drug outlets to take up MRDTs, and all those available were conducted in Uganda. One such study [23] noted similar findings: most drug retailers were not previously familiar with MRDTs, but after the explanation or demonstration of the product, they felt it would be useful to them and beneficial to sell in their shops. Retailers in both studies felt that MRDTs could increase confidence in a malaria diagnosis and treatment, bring business to their shops, and improve their reputation as medicine retailers. Both studies' findings underscore the potential for MRDTs to be accepted in the retail environment, and to have a positive impact on diagnostic access. This is found to be an untapped area of possibility, as most medicine retailers were unfamiliar with rapid diagnostic tests. 

Focus group participants also anticipated concerns of their clients. Similar to the Ugandan study by Mbonye and colleagues [24], shop workers in Webuye expressed concern that clients may refuse to use MRDTs out of fear of being tested for HIV. We found that retailers held perceptions that their customers may not accept a nonmalaria diagnosis or may turn to witchcraft or other spiritual explanations for illness that was not caused by malaria. These concerns highlight the importance of community education and awareness to reduce misconceptions about the function and use of rapid diagnostic tests for malaria as well as education regarding alternative causes of fever that are symptomatically similar to malaria. 

Adherence to the results of a rapid test for malaria, particularly a negative result, remains a concern given the findings of recent implementation studies in health facilities. One such study in public dispensaries in Tanzania [8] showed that health workers perceived MRDTs to be quick, easy to use, enhance appropriate treatment, increase confidence in diagnoses, and unanimously trusted by dispensary providers. However, the MRDT results did not always influence provider behavior appropriately, and often antimalarials were prescribed to customers with a negative MRDT; at the start of the implementation, roughly 30% of children who were given antimalarials had a negative test. Similar results were seen in Uganda [12] where 35% of those who tested negative by MRDT in a health facility still received an antimalarial. 

In a retail outlet, where healthcare consumers are not patients but are clients, adherence to the test result may be more influenced by the preferences of the customer and the financial incentives faced by providers. Customers may not accept test results and may demand antimalarials. Shop workers may fear losing business if they do not respond to customer demands. 

Shop workers themselves may also reject the results of the test or fail to offer a test to the customer. Our study found several shop workers who described scenarios in which they may not trust test results, potentially leading to an underutilization of the tests. A recent study by Kedenge and colleagues [31] evaluating the effectiveness of a subsidized antimalarial intervention in Kenyan retail shops found that shop workers did not consistently perform certain components of the intervention such as provision of advice, stating that they were too busy with other customers. This trend among for-profit shop workers may limit their ability to accurately and consistently administer rapid tests to their clients. Educating and training retailers on the sensitivity and specificity of the MRDT, building trust in the test results, demonstrating the negative effects of dispensing antimalarials to someone sick with another disease, and coaching retailers on how to respond to a client with a negative drug test can also help increase implementation of MRDTs and adherence to test results.

Adherence to the test results is not the only barrier to safe and correct implementation of MRDTs in retail outlets. Training must include instruction on blood handling and safe disposal of used lancets and other materials. Proper use of the kit, including loading the blood, waiting for the results, and interpreting the results, has been identified as occasionally problematic in studies training laypersons to use MRDTs. However, proper use of MRDTs has been achieved in community health worker programs [32–35] demonstrating that these problems are not insurmountable, but implementation of MRDTs may require more supervision and oversight than is currently provided in the retail sector. 

The regulatory environment and health system context were key barriers identified by focus group participants that need to be addressed prior to implementation. Participants in Uganda [24] discussed concerns that health workers and district health officials felt that drug shops were inadequately supported by the government and often were not included in education opportunities. The findings from this study point more to concerns that the regulatory agencies responsible for regulation of retail drug shops may not approve of their providing diagnostic services. In a study by Chandler and colleagues [23], retail drug shops were viewed with suspicion and assumed to often operate outside the law. This perception, whether founded or unfounded, can affect MRDT implementations due either to a restrictive regulatory environment or a mistrusting clientele. The majority of retail shops selling AL are unregistered and sell AL illegally over the counter. Given the restrictive but largely unenforced regulatory environment in Kenya, implementing MRDTs in this area may hold different challenges than were experienced in Cambodia or other regions. Governmental and regulatory policies are concerns that are shared in both regions, but the regulatory environments are quite different and should be addressed locally. 

While these focus group discussion findings provide valuable insight into the perceptions medicine retailers hold regarding the sale and use of MRDTs, they are limited in that participants may have shared what they believe is the correct views, or views expected by study staff, rather than their actual opinions. Most participants were not familiar with RDTs and, following the demonstration, their comments may have partly been in response to the novelty of the tests rather than their expected utility. Pilot implementation studies may therefore shed additional light on true acceptability of malaria testing in a retail setting. 

There was found an inconsistency in the type of health training and level of education collected by the brief questionnaire administered before the start of the focus group discussions. Forty-five percent of participants indicating a pharmacist training (5/11), and 47% of participants indicating a nursing or midwifery training (8/17) reported that they did not have an education above secondary school. Additionally, two participants reported their level of health-related qualification to be that of clinical officer, but one of these reported that they had only received education through primary school. However, to hold diploma certificates in these training areas requires an education above secondary school. This inconsistency could be explained by either the participants misunderstanding the question pertaining to health training or education on the survey or that they self-identify with a certain health title that comes from holding that occupation, rather than from having formal training. A similar dichotomy was found in a recent quantitative investigation that studied a similar population [25]. Following discussions on this finding with local research team, clinicians, and public health practitioners familiar with the area, it was their opinion that medicine retailers, particularly in rural areas, are referred to by the local community as “pharmacists” or “nurses” because these are the social roles they fill in the community, not bearing on whether those individuals hold official health qualifications in that field. It was conjectured that these retailers may self-identify in accordance with these roles. 

Since the study did not collect personal information about the participants, and did not require advance identification of participants discussion comments could not be sorted by gender, training, or type of retail facility. As a result, having men and women in the same discussions, or high and low levels of training represented in the same discussion, may have inhibited some participants from speaking or from sharing truthful answers. This study did not include consumer perceptions, which could also influence the feasibility of implementing MRDTs in this environment. Further research needs to be conducted in this area to ensure that retailers are acceptable providers of diagnostic services, and that perceptions consumers have regarding the purchase and use of MRDTs are well known and accounted for in intervention planning. 

## 5. Conclusions and Recommendations

Given the established consumer behavior of first seeking malaria treatment from medicine retailers, and the high rates of overdiagnosis of malaria, the introduction of MRDTs in these medicine outlets has the potential to substantially improve appropriate treatment. Studies have shown that MRDTs are highly sensitive and specific for malaria, and that withholding antimalarials in cases with negative tests does not put children at greater risk for complication or death [26, 36, 37]. Implementing MRDTs in retail locations is an important next step in increasing access to much-needed diagnostic services.

However, this study uncovered potential challenges to implementation that should be addressed in advance of these efforts. Addressing retailer concerns regarding community acceptability by heightening community awareness of the potential for nonmalaria causes of fever will be critical to creating an environment where medicine retailers can adhere to MRDT results and withhold antimalarials in cases with negative tests. Support from local community leaders, chiefs, and elders, as well as from private health sector consumers, public health facilities, and regulatory agencies, will assist in developing a solid foundation for the implementation of MRDTs. 

Pilot studies are now underway exploring the use of MRDTs in private outlets [38, 39]. Results from such studies need to be carefully examined to measure the wider impact of providing testing in drug outlets, and how this provision relates to or may impact the greater healthcare delivery system, the supplier/consumer relationship, treatment-seeking behavior, and the relationship between the informal and formal healthcare sectors. 

## Figures and Tables

**Table 1:** Focus group participant data from self-administered questionnaire.

	Peri-urban			Rural						
	FGD 1	FGD 2		FGD 3	FGD 4	FGD 5	FGD 6		Total	*% of total *
Total participants	5	5		8	10	16	17		61	*100 *
Women	4	2		5	6	7	12		36	*59 *
Men	1	3		3	4	9	5		25	*41 *
Age								
Under 20	1	0		0	0	1	0		2	*3 *
21–30	1	4		1	4	2	8		20	*33 *
31–40	0	1		4	3	7	5		20	*33 *
41–50	1	0		1	1	2	2		7	*11 *
Over 50	1	0		1	1	1	0		4	*7 *
*Unreported *	*1 *	*0 *		*1 *	*1 *	*3 *	*2 *		*8 *	*13 *
Training								
Pharmacist	0	1		1	2	3	4		11	*18 *
Pharmacy tech	1	1		0	1	2	0		5	*8 *
Pharmacy asst	1	1		2	0	4	3		11	*18 *
Medical doctor	0	0		0	0	0	0		0	*0 *
Nurse/midwife	1	0		2	3	5	6		17	*28 *
Clinical officer	0	0		0	0	1	1		2	*3 *
Untrained	1	0		0	0	0	0		1	*2 *
*Unreported *	*1 *	*2 *		*3 *	*4 *	*1 *	*3 *		*14 *	*23 *
Education								
Completed primary	0	0		0	0	0	1		1	*2 *
Some secondary	0	0		0	1	1	2		4	*7 *
Completed secondary	4	0		6	3	4	8		25	*41 *
Above secondary	0	3		1	4	11	5		24	*39 *
*Unreported *	*1 *	*2 *		*1 *	*2 *	*0 *	*1 *		*7 *	*11 *
